# Collagen Peptide Exerts an Anti-Obesity Effect by Influencing the Firmicutes/Bacteroidetes Ratio in the Gut

**DOI:** 10.3390/nu15112610

**Published:** 2023-06-02

**Authors:** Ga Hyeon Baek, Ki Myeong Yoo, Seon-Yeong Kim, Da Hee Lee, Hayoung Chung, Suk-Chae Jung, Sung-Kyun Park, Jun-Seob Kim

**Affiliations:** 1 Department of Nano-Bioengineering, Incheon National University, Incheon 22012, Republic of Korea; inu202221153@inu.ac.kr; 2 Infectious Disease Research Center, Korea Research Institute of Bioscience and Biotechnology (KRIBB), Daejeon 34141, Republic of Korea; we8536@kribb.re.kr (K.M.Y.); duddl765@gmail.com (S.-Y.K.); remake91@naver.com (D.H.L.); 3 Department of Functional Genomics, KRIBB School of Bioscience, Korea University of Science and Technology (UST), Daejeon 34113, Republic of Korea; 4 Department of Plant Resources, College of Industrial Science, Kongju National University, Yesan 32439, Republic of Korea; hayoung0713@gmail.com; 5 Sempio Fermentation Research Center, Sempio Foods Company, Cheongju 28156, Republic of Korea; jschea@gmail.com

**Keywords:** collagen peptide, intestinal microbial flora, prebiotics, Firmicutes/Bacteroidetes ratio, anti-obesity effect

## Abstract

Alterations in the intestinal microbial flora are known to cause various diseases, and many people routinely consume probiotics or prebiotics to balance intestinal microorganisms and the growth of beneficial bacteria. In this study, we selected a peptide from fish (tilapia) skin that induces significant changes in the intestinal microflora of mice and reduces the Firmicutes/Bacteroidetes ratio, which is linked to obesity. We attempted to verify the anti-obesity effect of selected fish collagen peptides in a high-fat-diet-based obese mouse model. As anticipated, the collagen peptide co-administered with a high-fat diet significantly inhibited the increase in the Firmicutes/Bacteroidetes ratio. It increased specific bacterial taxa, including *Clostridium_sensu_stricto_1, Faecalibaculum, Bacteroides*, and *Streptococcus*, known for their anti-obesity effects. Consequently, alterations in the gut microbiota resulted in the activation of metabolic pathways, such as polysaccharide degradation and essential amino acid synthesis, which are associated with obesity inhibition. In addition, collagen peptide also effectively reduced all obesity signs caused by a high-fat diet, such as abdominal fat accumulation, high blood glucose levels, and weight gain. Ingestion of collagen peptides derived from fish skin induced significant changes in the intestinal microflora and is a potential auxiliary therapeutic agent to suppress the onset of obesity.

## 1. Introduction

Chemical compounds are typically screened for their potential use as medicines for specific ailments. During the early screening stages, millions of compounds were evaluated against the processes or phenotypes of diseases. However, this initial process is time- and cost-intensive and poses a risk of failure in the downstream process owing to cytotoxicity, permeability, and a variety of other characteristics. On average, only large pharmaceutical corporations are willing to accept this risk. To address this, novel screening methods that are both rapid and promising are required for disease treatments. Here, we conducted a gut microbiota-based screening strategy to select nutritional supplements as prebiotic medications.

The relationship between human diseases and gut microbiota has been extensively studied over the last two decades. The human intestine contains 3.3 million microbial genes, 150 times the number found in the human genome, and is known to harbor over 1000 different bacteria [1]. Research on the interaction between the gut microbiome and human health has been aggressively conducted. Changes in the composition and function of gut bacteria have been associated with inflammatory bowel disease, Crohn’s disease, and ulcerative colitis [2,3,4,5]. Additionally, numerous pieces of evidence point to gut microbiota perturbations as a cause of atopic asthma, type 2 diabetes, autoimmune diseases, and even behavioral disorders [6,7,8,9,10,11]. This reflects that the consumption of probiotics or prebiotics has more value as a therapeutic agent than just a nutritional supplement. Therefore, in addition to the traditional method of screening therapeutic agents that act directly on diseases, selecting compounds that affect the gut microbiota is a new worthwhile therapeutic development platform.

Unlike probiotics that directly involve live microorganisms, prebiotics refer to ‘substrates’ that are selectively utilized by host microorganisms conferring a health benefit [12]. Therefore, we assessed the possibility of using peptides extracted from food as prebiotics by linking them to changes in the composition of intestinal microbes. To facilitate absorption into the body and minimize side effects, edible low-molecular-weight peptides were randomly selected and orally administered to mice.

Based on the results of gut microbiota alteration mediated by peptides, collagen-derived peptides and obese mouse models were selected. Since collagen peptide consumption led to a significant reduction in the Firmicutes/Bacteroidetes (F/B) ratio in normal mice, its anti-obesity effect was expected. Early research on the relationship between obesity and gut microbiota established that significant fractions of intestinal *Bacteroidetes* and *Firmicutes* are altered in obese mice compared with those in lean mice [13]. The same group also demonstrated that germ-free mice transplanted with microbiota from an obese donor showed a significantly higher body fat percentage than mice transplanted with microbiota from a lean donor [14]. These findings provide compelling evidence that gut microbiota remodeling can regulate microbiota-associated disease symptoms. Using an obesity-induced mouse model, the anti-obesity effect of collagen peptide consumption has been confirmed in various ways, including weight gain, blood tests, body fat accumulation, and NGS. These indicate that a microbiota-based screening system of edible peptide works. Based on our results, the study of changes in the gut microbiota appears to be of sufficient value as an alternative drug screening system for directly edible substances.

## 2. Materials and Methods

### 2.1. Preparation of Fish-Collagen Peptides

To eliminate contaminants from the skin of fish (tilapia), it was consecutively treated with a basic mixture of NaOH, Na_2_SO_4_, and NaHCO_3_ equivalent to 1 N and an acidic mixture of HCl and H_2_SO_4_ corresponding to 1 N. Then, a sterilization method was used to prepare a pretreatment marine skin sample. A prepared sample of marine skin was treated with a mixture of endopeptidase and exopeptidase enzymes at a concentration of 2% by weight of raw material. Following treatment, collagen peptides obtained from fish were purified sequentially using decolorization and deodorization, as well as micro-, ultra-, and nano filter systems (10-1071338). The molecular weight and amino acids compositions are provided in Tables S1 and S2.

### 2.2. Animals, Diets, and Peptide Treatment

All mice were housed in a specific pathogen-free facility at the Korea Research Institute of Bioscience and Biotechnology (KRIBB, Daejeon, Korea). The mice were maintained under a 12 h light-dark cycle at 23 °C and fed a standard diet with water ad libitum. Seven-week-old male C57BL/6 mice were acclimated for 1 week before random separation into experimental groups. All mice were orally administered 100 μL of distilled water (DW) or peptide solution in DW by gavage. Regarding the high-fat diet (HFD), the mice were fed a 60 kcal % fat diet (Research Diet, D12492) instead of the standard diet. Body weight changes for each mouse and the amount of food consumed by all mice were monitored every 3 or 4 days during the experimental period.

### 2.3. Collection of Blood and Fecal Samples

On day 21 after the HFD, blood and fecal samples were collected from all mice in the presence or absence of peptide treatment. Blood was drawn from the retro-orbital sinus of each mouse and centrifuged (3000 rpm for 20 min at 4 °C) to separate the serum. The blood biochemical analyses were performed using a K-Bio (Osong Medical Innovation Foundation, Osong, Korea). To collect the fecal samples, the large intestine was quickly removed from the mice after euthanasia and extracted by squeezing longitudinally. In addition, feces excreted from all mice were collected using sterile forceps every 3 or 4 days per week. The fresh feces were stored at −80 °C immediately after collection for the microbiota and endotoxin analyses.

### 2.4. Fecal Endotoxin Assay

The fecal endotoxin content was determined using the ToxinSensor^TM^ Chromogenic Limulus amebocyte lysate (LAL) Endotoxin Assay Kit (GenScript, Piscataway, NJ, USA, L00350) according to the manufacturer’s protocol. Briefly, an appropriate amount (1 μg) of feces from each mouse was placed in 1 mL of phosphate-buffered saline (PBS) and subjected to Bioruptor sonication (Diagenode) for 15 cycles with 30/30 s On/Off. After centrifugation at 400× *g* for 15 min, the upper solution was passed through a 0.45 μm syringe filter and then inactivated for 10 min at 70 °C. Next, 25 μL of the sterilized fecal solution was used for the LAL assay, and the absorbance of each reaction was measured at 545 nm using a SpectraMax 190 Microplate Reader (Molecular Devices).

### 2.5. Cell Culture

A murine macrophage cell line, RAW264.7 (ATCC, TIB-71) was maintained at 37 °C in Dulbecco’s modified Eagle’s medium (DMEM) supplemented with 10% heat-inactivated fetal bovine serum (FBS; Corning) and 1× antibiotic-antimycotic (ThermoFisher Scientific, Waltham, MA, USA, 15240112) in an atmosphere of 5% CO_2_ saturated with water. To examine inflammatory cytokine induction by stool lysate from mice, 65 μL of the sterilized fecal solution, prepared for the endotoxin assay, was applied to RAW264.7 cells for 24 h.

### 2.6. ELISA

Both the mouse IL-6 (BioLegend, San Diego, CA, USA, 43101) and TNF-α (BioLegend, 430901) in RAW264.7 cell culture media incubated with fecal lysate were measured using ELISA kits following the manufacturer’s instructions. In brief, half-area microplates (Corning) were coated with 50 µL of each capture antibody solution and incubated overnight at 4 °C. The wells were washed thrice with PBS plus 0.05% Tween 20 and blocked for 1 h at 37 °C with 100 µL of blocking buffer in the kit. Next, the wells were washed thrice and incubated with 50 µL of each sample for 2 h at room temperature. After washing thrice, the wells were incubated with 50 µL of each detection antibody solution for 1 h at room temperature, washed again, and incubated with 50 µL streptavidin-HRP for 30 min at room temperature in the dark. Finally, the wells were washed five times and incubated with 50 µL of the substrate solution for 15 min at room temperature in the dark. After color development, 25 µL of 2 M H_2_SO_4_ was added to stop the reaction, and absorbance was measured at 450 nm using a SpectraMax 190 Microplate Reader (Molecular Devices).

### 2.7. Gut Microbiome Analysis

Using the QIAamp PowerFecal Pro DNA kit (Qiagen, 51804), fecal DNAs were extracted. The V3V4 sections of the 16s rRNA gene were amplified with the following primers: 341F-TCGTCGGCAGCGTCAGATGTGTATAAGAGACAGCCTACGGG-NGGCWGCAG and 805R-GTCTCGTGGGCTCGGAGATGTGTATAAGAGACAG-GACTACHVGGGTATCAATTCC. The amplicons were sequenced utilizing the MiSeq platform (Illumina). The contigs were created from paired-end reads, data cleaning, sequence alignment, taxonomic classification, and clustering in OTUs. The diversity analyses were performed using Mothur v.1.47.0 and the SILVA reference database (http://www.mothur.org/) [15]. The distance between the gut microbiome in each sample was calculated using Jaccard coefficients and visualized using PCoA [16]. Statistical significance also evaluated using the analysis of molecular variance (AMOVA) on Mothur v.1.47.0. The alpha diversity was measured using indices of community richness (Chao and Sobs) and community diversity (Shannon and Invsimpson) [17]. All the statistical analyses were performed using GraphPad Prism version 7.0 (GraphPad Software, SanDiego, CA, USA), and the significance level used for all the tests was *p* < 0.05. LEfSe was used to analyze OTUs with statistically significant differences between the two groups [18]. The cutoff value was an absolute LDA score (log 10) > 2.0. The functional capacity of the gut microbiome was predicted using the PICRUSt2 in the MetaCyc pathway, and visualized using the R package pheatmap v1.0.12 [19,20]. STAMP version 2.1.3 was used to identify differentially metabolic pathways [21].

## 3. Results

### 3.1. Screening of Valuable Peptides for Disease Treatment Based on the Analysis of Gut the Microbiota Composition

**Figure 1:**
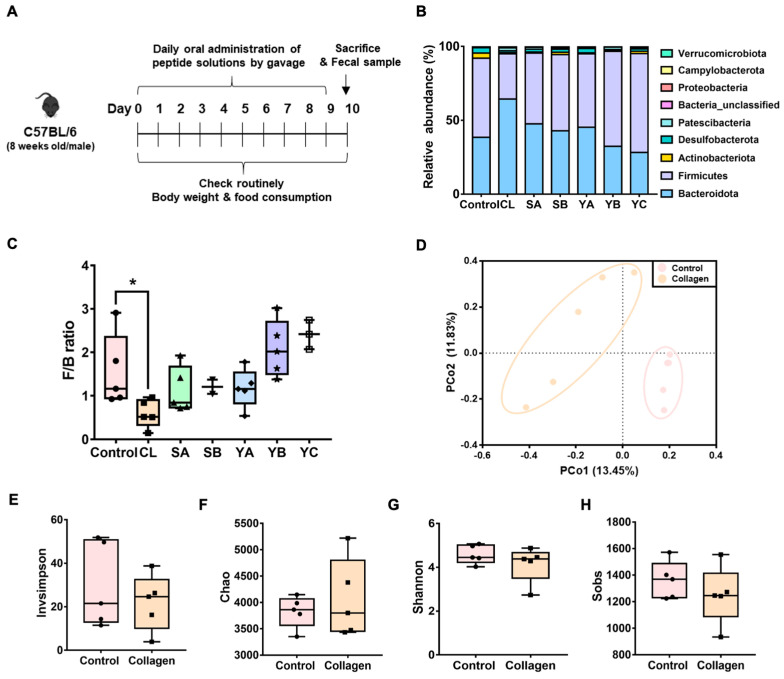
Screening of peptides with a significant effect on the intestinal microflora. (**A**) The schematics of the animal study (C57BL/6) design. From days 0–9, the peptide solutions were administered to mice by oral gavage. On day 10, the mice were sacrificed, and the fecal matter was sampled. (**B**) The relative abundance of the gut microbiota at the phylum levels in six peptide-gavaged mice. The nine most abundant bacteria phyla were obtained from 30 mouse fecal samples from seven groups of mice. (CL; collagen, SA; soybean type A, SB; soybean type B, YA; yeast type A, YB; yeast type B, YC; yeast type C). (**C**) Firmicutes/Bacteroidetes (F/B) ratio. Unpaired *t*-tests (two-tailed) were used to analyze variations between the two groups. * *p* < 0.05. (**D**) Beta diversity. The PCoA was based on the Jaccard distance, and the principal coordinate with the largest contribution rate was selected for graphical display. The two groups were completely separated. The statistical analyses were performed using AMOVA. (**E**–**H**) Alpha diversity. Sobs, Shannon, Invsimpson, and Chao indices reflect the differences in richness and evenness among samples.

To conduct this screening method, we first selected six peptides from fish collagen (CL), soybean type A, B (SA, SB), and yeast type A, B, C (YA, YB, YC). The influence of peptides on the gut microbiota composition was evaluated by daily administration of peptides solubilized in sterile distilled water to mice via gavage for 10 days. For the gut microbiota analyses, feces were collected on days 3 and 7 after peptide treatment, and the intestines were isolated from sacrificed mice on day 10 (Figure 1A). The 16S rRNA gene amplicon sequence analysis using fecal samples from mice revealed that Bacteroidetes and Firmicutes accounted for the majority (~90%) of the total microbiota in all treatment groups. Collagen peptide treatment for 10 days increased the abundance of Bacteroidetes to 64% and decreased Firmicutes to 31% in the gut compared with the water treated control group (38% and 54%, respectively) (Figure 1B). Interestingly, compared with the control group, fish collagen-derived peptides significantly decreased the F/B ratio, whereas other peptides did not affect this ratio (Figure 1C). The principal coordinate analysis (PCoA) using the Jaccard distance metric clearly showed a separation between the two groups that is significant via AMOVA (Figure 1D, *p* < 0.005). However, the alpha diversity analysis revealed no significant differences in the richness or evenness of the collagen peptide-treated and control groups (Figure 1E–H). In addition, considering that the change in the F/B ratio in the gut was already confirmed three days after the administration of the collagen peptide (Figure S1), its influence on the gut microbiota composition seemed to have appeared immediately after ingestion. Collectively, these results demonstrated that collagen peptide consumption causes alterations in the gut microbiota in the direction of a lower F/B ratio.

### 3.2. Verification of Anti-Obesity Effects of Collagen Peptides Screened by Gut Microbiome Analysis

**Figure 2:**
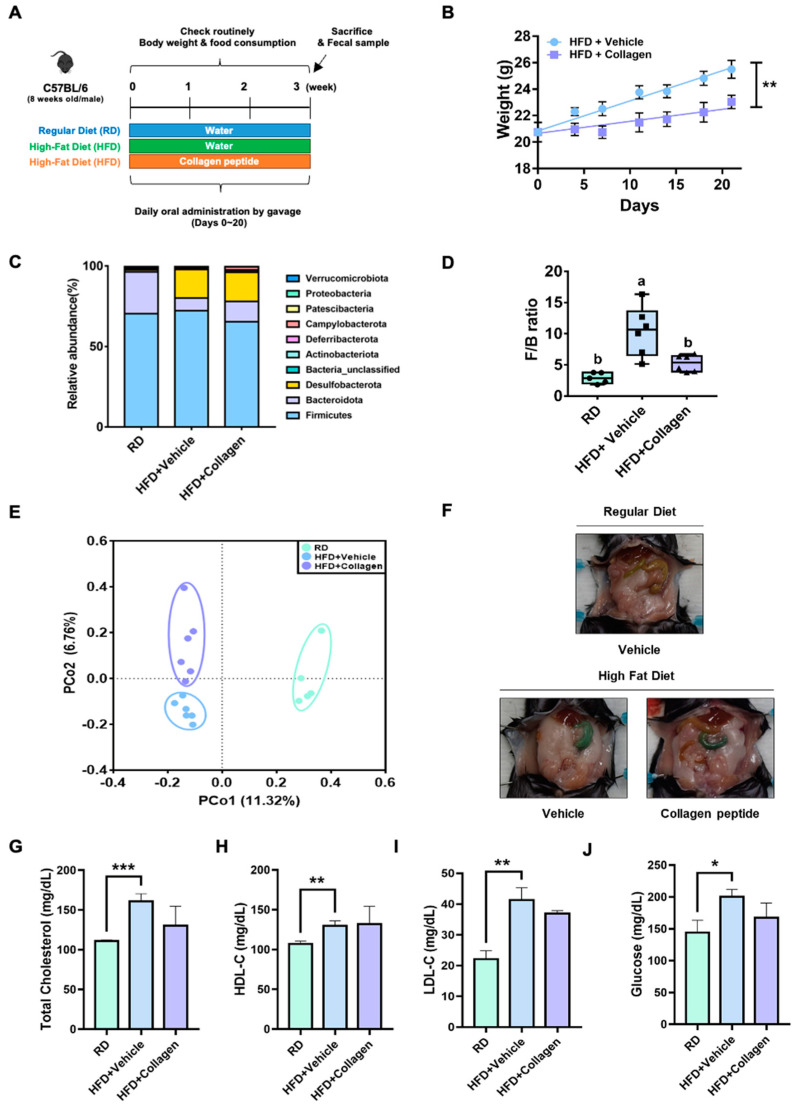
The anti-obesity effects of collagen peptide screened by gut microbiome analysis. (**A**) The schematics of the animal study (C57BL/6) design (High-fat diet model). We administered collagen peptide in the HFD mouse model by oral gavage for three weeks and monitored the body weight and food consumption. After three weeks, we sacrificed the mice and sampled the fecal matter. (**B**) Daily weight of the HFD mouse model in the presence or absence of collagen peptide. The HFD mice administered collagen peptides daily gained less weight than mice fed with HFD alone. Unpaired *t*-tests (two-tailed) were used to analyze variations between the two groups. ** *p* < 0.01. (**C**) The relative abundance of the gut microbiota at the phylum level in RD, HFD + vehicle, and HFD + collagen mice. The 10 most abundant bacterial phyla were obtained from 17 mouse fecal samples from three groups of mice. (**D**) Firmicutes/Bacteroidetes (F/B) ratio. The one-way ANOVA test was used to analyze variation between the three groups. Different letters indicate statistical significance. (**E**) Beta diversity. PCoA was based on the Jaccard distance, and the principal coordinate with the largest contribution rate was selected for graphical display. The three groups were completely separated. Statistics analyses were performed using AMOVA. (**F**) Abdominal body fat comparison in the three groups. The decrease in abdominal body fat of HFD mouse model in the presence of collagen peptide was evident. (**G**–**J**) The results of the blood-biochemical analysis. Cholesterol or glucose levels were increased significantly in the mice fed with HFD for three weeks compared with the RD mice. Unpaired *t*-tests (two-tailed) were used to analyze variations between the two groups. * *p* < 0.05, ** *p* < 0.01, *** *p* < 0.001.

Since it is well known that the F/B ratio in the gut of obese mice is abnormally increased compared with normal or lean mice, our 16S rRNA gene sequencing results suggested that collagen peptides may have anti-obesity properties by affecting the F/B ratio of the gut microbiota. In a mouse model with a high-fat diet (HFD), the correlation between the F/B ratio in the gut microbiota and body weight change according to the presence or absence of collagen peptide treatment, was determined (Figure 2A). In the absence of collagen peptide treatment, the body weight of mice fed a HFD for three weeks was significantly increased compared with that of mice fed a regular diet (RD) (Figure S2A). However, HFD mice administered collagen peptides daily gained less weight than mice fed HFD alone (Figure S2A and Figure 2B). Compared with food intake for three weeks, there was no significant weight change in the presence or absence of peptide treatment (Figure S2B), suggesting that the administration of collagen peptide inhibited weight gain caused by HFD other than food consumption. In our model, HFD resulted in a substantial increase in Desulfobacterota and a significant decrease in Bacteroidetes in the intestine of mice (Figure 2C), which is consistent with the outcome of a previous study [22]. Additionally, the F/B ratio of HFD mice with collagen treatment was substantially reduced compared with that of HFD-alone mice (Figure 2D). Next, we compared the microbial community composition (beta diversity) of the three groups using PCoA of Jaccard distances. The collagen peptide consumption revealed a clear distinction in the gut microbiota composition compared with HFD alone (Figure 2E), leading to a decrease in the intestinal F/B ratio of obese mice and consequently inhibiting body weight gain caused by the HFD. In addition, significant differences were observed in the gut microbiota richness and evenness (alpha diversity) among the groups (Figure S3).

Blood biochemical analysis revealed that cholesterol or glucose levels were increased significantly in the mice fed with HFD for three weeks compared with that of RD mice, showing a similar pattern to weight gain (Figure 2G–J). Co-administering the collagen peptide to mice fed with HFD showed a trend toward substantial decrease in blood cholesterol and glucose levels, but this was not statistically significant. In addition, reduced abdominal body fat was evident (Figure 2F). Given the positive correlation between the body fat percentage and blood glucose levels, these results were consistent with those of a previous study showing that the body weight changes between obese and lean mice, according to the alteration in the F/B ratio of the gut microbiota, was dependent on the accumulation of body fat [14]. In other words, collagen peptide treatment showed an anti-obesity effect, presumably by reducing body fat mass increased by HFD by influencing the F/B ratio of the intestinal microbes.

### 3.3. Differential OTUs of the Gut Microbiota by Collagen Peptide Consumption with HFD

**Figure 3:**
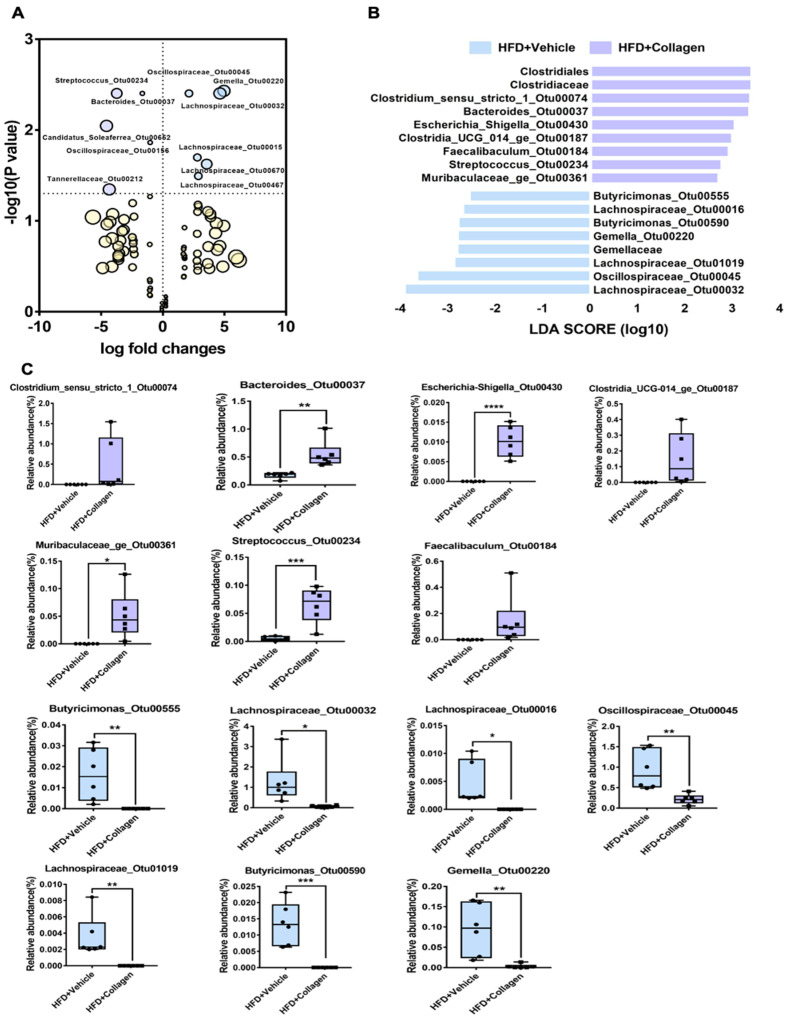
The difference of OTU in two groups (HFD + vehicle and HFD + collagen). (**A**) Log fold changes. The log2 (vehicle/collagen) is the ratio of the relative abundance of OTUs between the two groups. As the size increases, it means that the relative abundance of the corresponding OTU is high. Fourteen distinguishing taxa (OTUs) were significantly marked among 50 differential OTUs between the two groups. (**B**) Differentially abundant bacterial taxa in fecal samples from mice models (HFD + vehicle and HFD + collagen). A bar plot showing the LDA score (effect size) of differentially abundant OTUs in the HFD + vehicle (blue, *n* = 6) and HFD + collagen (purple, *n* = 6) groups as determined using the Linear Discriminant Effect Size (LEfSe) analysis (α = 0.05, LDA score > 2.0). (**C**) A box plot comparing the relative abundance of OTUs between the two groups. Unpaired *t*-tests (two-tailed) were used to analyze variation between the two groups. * *p* < 0.05, ** *p* < 0.01, *** *p* < 0.001, and **** *p* < 0.0001.

Based on differential abundances, 11 distinct taxa (OTUs) were marked among the top 50 differential OTUs between the groups. Consumption of collagen peptides with HFD significantly increased five bacterial taxa: Streptococcus (OTU00234), Bacteroides (OTU00037), Candidatus Soleaferrea (OTU00662), Oscillospiraceae (OTU000156), and Tannerelaceae (OTU00212). However, collagen peptide significantly decreased six bacterial taxa, including Oscillospiraceae (OTU00045), Gemella (OTU00220), and Lachnospiraceae (OTU00032, OTU00015, OTU00670, and OTU00467) (Figure 3A).

The linear discriminant analysis effect size (LEfSe) analysis was employed to examine the particular bacteria linked to collagen peptide ingestion by screening the distinct gut microbiota indicators in each group. Nine taxa were newly identified by linear discriminant analysis (LDA) scores above 2.0. These included Clostridium_sensu_stricto_1 (OTU00069), Escherichia-Shigella (OTU00449), Clostridia_UCG_014 (OTU00187), Faecalibaculum (OTU00184), Muribaculaceae (OTU00361), Butyricimonas (OTU00555, OTU00590), and Lachnospiraceae (OTU00016 and OTU01019) (Figure 3B). Particularly, Clostridium_sensu_stricto_1, Muribaculaceae, Faecalibaculum, and Escherichia-Shigella were significantly enriched by collagen peptide consumption; however, these were not found in the gut of HFD mice. In addition, Butyricimonas and Lachnospiraceae were eliminated by collagen peptide consumption (Figure 3C and Figure S4).

### 3.4. Alteration of the Gut Microbiota Mediated by Collagen Peptide Consumption Shifts the Microbiota-Mediated Metabolic Significance

**Figure 4:**
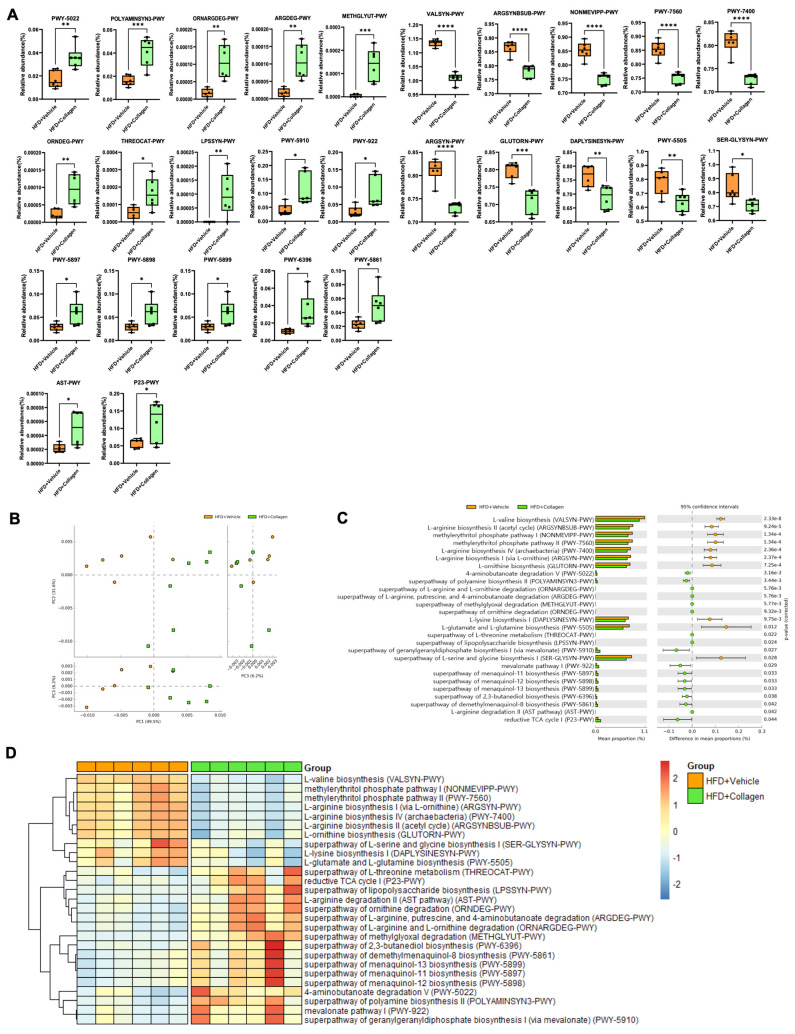
The predicted functional potential changes between HFD + vehicle mice and HFD + collagen mice by using PICRUST2. (**A**) The boxplots showed the relative abundance in 27 pathways related to obesity. There were 17 significantly increased pathways and 10 decreased pathways in HFD + collagen compared to HFD + vehicle. (**B**) The PCA analysis of intestinal bacterial metabolic pathway, the orange color indicates HFD + vehicle mice and the green color indicates HFD + collagen mice. (**C**) The extended error bar chart showed the significant difference in the predicted functional pathways related to obesity between the two groups. The middle value represents the mean differences between the two groups (upper-lower bar value), and the error bar represents the 95% confidence intervals with the effect size of difference in mean proportion. The *p*-value at the side indicates the significance between the upper and lower bars. (**D**) Heatmaps were drawn with normalized relative abundances in 27 obesity-related pathways in the two groups. The red colors represent a higher abundance and blue colors a lower abundance. * *p* < 0.05, ** *p* < 0.01, *** *p* < 0.001, and **** *p* < 0.0001.

To understand the metabolic signatures of the gut microbiota alteration mediated by collagen peptide consumption, phylogenetic investigation of the communities by reconstruction of unobserved states (PICRUSt) and MetaCyc-based analysis using 16S rRNA data were employed to determine the metabolic pathways with significant differences. Among the top 60 metabolic pathways with differences between the groups (Figure S6), 27 pathways related to obesity were identified based on previous studies (Figure 4). The relative abundance of secondary metabolite biosynthesis and amino acid biosynthesis was higher in the HFD group, whereas aldehyde degradation, lipopolysaccharide biosynthesis, cofactor, carrier, vitamin biosynthesis and proteinogenic amino acid degradation pathways were higher in the HFD + collagen group, which may be associated with obesity lesions.

### 3.5. HFD-Induced Fecal Endotoxin Levels Were Decreased by the Administration of Collagen Peptides

**Figure 5:**
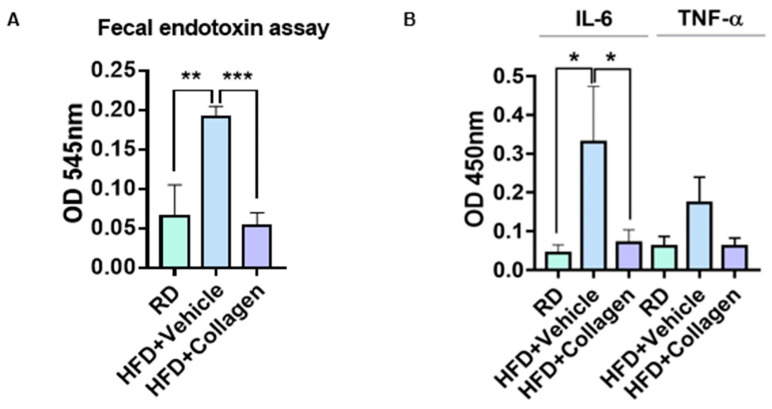
The inhibitory effect of collagen peptide on HFD-derived endotoxin excretion in the gut. (**A**) Endotoxin levels in the sterilized fecal solution were measured by Limulus amebocyte lysate (LAL) assay. (**B**) Murine macrophage cell line, Raw264.7 cells, were incubated with a sterilized fecal solution for 24 h, and then pro-inflammatory cytokine levels in culture media were measured by ELISA. (**A**,**B**) Unpaired *t*-tests (two-tailed) were used to analyze the variation between the two groups. * *p* < 0.05, ** *p* < 0.01, *** *p* < 0.001.

It is widely accepted that an HFD-induced increase in F/B ratio causes decreased expression of short-chain fatty acids (SCFA), which maintain the intestinal epithelial barrier integrity by improving tight junction protein expression, resulting in severely elevated endotoxemia in the gut [23]. To investigate whether HFD-induced fecal endotoxin levels were reduced by collagen peptide treatment, we compared fecal endotoxin concentrations in the intestine in the presence and absence of peptide ingestion. As expected, fecal endotoxin levels were substantially increased by HFD compared with RD (Figure 5A). Co-administration of collagen peptide to mice fed with HFD reduced fecal endotoxin levels (Figure 5A). In addition, as increased intestinal endotoxin levels in HFD mice are known to activate inflammatory responses, we examined cytokine production by treating the macrophage cell line RAW264.7 with fecal lysate. First, fecal lysates collected from HFD mice showed increased expression of pro-inflammatory cytokines, such as IL-6 and TNF-α, compared with RD (Figure 5B). However, when the fecal lysate from HFD mice orally administered with collagen peptides was applied to RAW264.7, the cytokine production induced by HFD decreased to normal levels (Figure 5B). These results indicate that collagen peptides may alleviate HFD-induced intestinal inflammatory responses by controlling intestinal endotoxin levels through an effect on the F/B ratio of the intestinal microbes.

## 4. Discussion

In this study, we attempted to design a medication-screening technique based on changes in the gut microbiota by shifting our perspective. To validate this concept, mice were randomly fed with ingestible low-molecular-weight peptides generated from food-derived proteins. After collagen peptide treatment, the F/B ratio in the gut microbiota substantially decreased, whereas the other peptides did not affect the F/B ratio. The consumption of collagen peptides substantially modified the gut microbiota composition but not its diversity or evenness (Figure 1).

As a decrease in the F/B ratio can result in weight loss in obese mice, the anti-obesity effect of collagen peptides was investigated. The consumption of collagen peptides during HFD resulted in various anti-obesity effects (Figure 2). In addition to a low buildup of body fat and blood biochemical analysis, collagen peptide ingestion considerably prevented weight increase in mice while consuming a HFD. Collagen peptide ingestion altered the composition of the gut microbiota shifted by a HFD, including a decrease in the F/B ratio (Figure 3). In particular, collagen peptide ingestion increased the abundance of specific bacterial taxa, such as *Bacteroides*, *Streptococcus*, *Faecalibaculum*, and *Clostridium sensu stricto* 1, which have a known relationship with obesity. *Bacteroides* probiotics in obese mice increases branched-chain amino acid metabolism in brown adipose tissue, thereby inhibiting obesity [24]. In addition, the abundance of *Streptococcus* and *Clostridium sensu stricto* 1 have been relatively lower in obese patients and animal models than in normal populations [25,26]. Furthermore, it was assumed that lactic acid-producing bacteria, particularly *Faecalibaculum*, have anti-obesity effects [27]. Additionally, collagen peptide intake reduced the relative abundance of *Butyricimonas*, *Lachnospiraceae*, and *Gemella*. A decrease in the richness of *Butyricimonas* and *Lachnospiraceae* has been reported during anti-obesity treatment with nuciferine or fucoxanthin [28,29]. Notably, *Lachnospiraceae* inoculation of germ-free ob/ob mice resulted in significant increases in blood glucose levels and fat tissue weight [30]. *Gemella* is one of the most common genera in obese individuals [31]. The *Akkermansia muciniphila*, which is known as an anti-obesity species [32] and is commercially available, did not show significant differences with collagen peptide consumption (Figure S4). We concluded that ingestion of collagen peptides had anti-obesity effects by altering the abundance of taxa associated with obesity.

In addition to the taxonomic analysis, the taxonomic-based metabolic pathway prediction demonstrated the anti-obesity effects of collagen peptide ingestion (Figure 4). Higher levels of obesity-related pathways, including amino acid degradation, aldehyde degradation, cofactor, carrier, vitamin biosynthesis and lipopolysaccharide biosynthesis were observed in the HFD plus collagen group, indicating that collagen intake may exert an anti-obesity effect. L-arginine and L-ornithine are converted to polyamine putrescine, decomposed to 4-aminobutanoate, and converted to succinate, which is used in the TCA cycle (Tricarboxylic Acid cycle). The TCA cycle decreased in the obese mice fed a HFD and increased in the obese animals [33]. In addition, succinate, an intermediary of the TCA cycle, stimulated heat production in brown adipose tissue, and the injection of succinate into mice fed a HFD validated its weight gain inhibition effect [34]. L-threonine reduces fat accumulation by regulating lipid metabolism in obese mice [35]. Methylglyoxal (MG) is a highly reactive compound derived primarily from glucose and fructose metabolism and is associated with obesity-related pathologies [36]. This metabolite correlates with serum LDL cholesterol and triglycerides and is of particular importance in obesity [37]. Menaquinol is an enzyme that produces menaquinone (vitamin K2), which affects the reduction of body weight and abdominal and visceral fat [38]. LPS promotes adipocyte apoptosis, the development of crown-like structures, caspase-4/5/11 activation, and apoptosis in adipocytes [39]. Additionally, it inhibits preadipocyte development and adipogenesis in adipose tissues [40]. In conclusion, collagen peptide consumption may exert anti-obesity effects on metabolic pathways and taxonomic analyses.

## Data Availability

Raw sequence data from mouse fecal samples have been deposited in the NCBI SRA database under the Bioproject ID PRJNA949392 (https://www.ncbi.nlm.nih.gov/bioproject/PRJNA949392).

## Supplementary Materials

The following supporting information can be downloaded at: https://www.mdpi.com/article/10.3390/nu15112610/s1, Table S1. Distribution of molecular weight for collagen peptide; Table S2. Distribution of molecular weight for collagen peptide; Figure S1. Changes in F/B ratio in the gut microbiome over time. The change in the F/B ratio in the gut microbiome was confirmed from the 3rd day of collagen peptide administration; Figure S2. Comparison of body weights for 3 weeks in the three groups (A). Comparison of changes in daily food intake between the two groups for 3 weeks (B). Unpaired *t*-tests (two-tailed) were used to analyze variations between the two groups. *** *p* < 0.001; Figure S3. Alpha diversity. Sobs, Shannon, Invsimpson, and Chao indices reflect the differences in microbiota richness and evenness among samples. Unpaired *t*-tests (two-tailed) were used to analyze variation between the two groups. * *p* < 0.05, ** *p* < 0.01, *** *p* < 0.001, **** *p* < 0.0001; Figure S4. Comparison of relative abundance in the genus *Akkermansia* known to have anti-obesity effects. An unpaired *t*-test (two-tailed) was used to analyze group variations. There was no significant difference; Figure S5. Bubble plot of the average relative abundance of OTUs significantly differed between the two groups. Circle size indicated the relative abundance from 0 to 3.5%; Figure S6. Heatmap visualization of significantly different functional profiles inferred by PICRUST2 according to the presence or absence of collagen peptide. Heatmaps were drawn with normalized relative abundances in all the pathways in the two groups. Red colors represent higher abundance, and blue colors lower abundance.

## References

[1] Qin J., Li R., Raes J., Arumugam M., Burgdorf K.S., Manichanh C., Nielsen T., Pons N., Levenez F., Yamada T. (2010). A human gut microbial gene catalogue established by metagenomic sequencing. Nature.

[2] Sokol H., Pigneur B., Watterlot L., Lakhdari O., Bermudez-Humaran L.G., Gratadoux J.J., Blugeon S., Bridonneau C., Furet J.P., Corthier G. (2008). Faecalibacterium prausnitzii is an anti-inflammatory commensal bacterium identified by gut microbiota analysis of Crohn disease patients. Proc. Natl. Acad. Sci. USA.

[3] Schirmer M., Franzosa E.A., Lloyd-Price J., McIver L.J., Schwager R., Poon T.W., Ananthakrishnan A.N., Andrews E., Barron G., Lake K. (2018). Dynamics of metatranscription in the inflammatory bowel disease gut microbiome. Nat. Microbiol..

[4] Mar J.S., LaMere B.J., Lin D.L., Levan S., Nazareth M., Mahadevan U., Lynch S.V. (2016). Disease Severity and Immune Activity Relate to Distinct Interkingdom Gut Microbiome States in Ethnically Distinct Ulcerative Colitis Patients. mBio.

[5] Kudelka M.R., Hinrichs B.H., Darby T., Moreno C.S., Nishio H., Cutler C.E., Wang J., Wu H., Zeng J., Wang Y. (2016). Cosmc is an X-linked inflammatory bowel disease risk gene that spatially regulates gut microbiota and contributes to sex-specific risk. Proc. Natl. Acad. Sci. USA.

[6] Strati F., Cavalieri D., Albanese D., De Felice C., Donati C., Hayek J., Jousson O., Leoncini S., Renzi D., Calabro A. (2017). New evidences on the altered gut microbiota in autism spectrum disorders. Microbiome.

[7] Stokholm J., Blaser M.J., Thorsen J., Rasmussen M.A., Waage J., Vinding R.K., Schoos A.M., Kunoe A., Fink N.R., Chawes B.L. (2018). Maturation of the gut microbiome and risk of asthma in childhood. Nat. Commun..

[8] Qin J., Li Y., Cai Z., Li S., Zhu J., Zhang F., Liang S., Zhang W., Guan Y., Shen D. (2012). A metagenome-wide association study of gut microbiota in type 2 diabetes. Nature.

[9] Li Q., Han Y., Dy A.B.C., Hagerman R.J. (2017). The Gut Microbiota and Autism Spectrum Disorders. Front. Cell. Neurosci..

[10] Le Chatelier E., Nielsen T., Qin J., Prifti E., Hildebrand F., Falony G., Almeida M., Arumugam M., Batto J.M., Kennedy S. (2013). Richness of human gut microbiome correlates with metabolic markers. Nature.

[11] Cekanaviciute E., Yoo B.B., Runia T.F., Debelius J.W., Singh S., Nelson C.A., Kanner R., Bencosme Y., Lee Y.K., Hauser S.L. (2017). Gut bacteria from multiple sclerosis patients modulate human T cells and exacerbate symptoms in mouse models. Proc. Natl. Acad. Sci. USA.

[12] Gibson G.R., Hutkins R., Sanders M.E., Prescott S.L., Reimer R.A., Salminen S.J., Scott K., Stanton C., Swanson K.S., Cani P.D. (2017). Expert consensus document: The International Scientific Association for Probiotics and Prebiotics (ISAPP) consensus statement on the definition and scope of prebiotics. Nat. Rev. Gastroenterol. Hepatol..

[13] Ley R.E., Backhed F., Turnbaugh P., Lozupone C.A., Knight R.D., Gordon J.I. (2005). Obesity alters gut microbial ecology. Proc. Natl. Acad. Sci. USA.

[14] Turnbaugh P.J., Ley R.E., Mahowald M.A., Magrini V., Mardis E.R., Gordon J.I. (2006). An obesity-associated gut microbiome with increased capacity for energy harvest. Nature.

[15] Schloss P.D., Westcott S.L., Ryabin T., Hall J.R., Hartmann M., Hollister E.B., Lesniewski R.A., Oakley B.B., Parks D.H., Robinson C.J. (2009). Introducing mothur: Open-source, platform-independent, community-supported software for describing and comparing microbial communities. Appl. Environ. Microbiol..

[16] McArdle B.H., Anderson M.J. (2001). Fitting multivariate models to community data: A. comment on distance-based redundancy analysis. Ecology.

[17] Hill T.C., Walsh K.A., Harris J.A., Moffett B.F. (2003). Using ecological diversity measures with bacterial communities. FEMS Microbiol. Ecol..

[18] Segata N., Izard J., Waldron L., Gevers D., Miropolsky L., Garrett W.S., Huttenhower C. (2011). Metagenomic biomarker discovery and explanation. Genome Biol..

[19] Langille M.G., Zaneveld J., Caporaso J.G., McDonald D., Knights D., Reyes J.A., Clemente J.C., Burkepile D.E., Vega Thurber R.L., Knight R. (2013). Predictive functional profiling of microbial communities using 16S rRNA marker gene sequences. Nat. Biotechnol..

[20] Caspi R., Altman T., Billington R., Dreher K., Foerster H., Fulcher C.A., Holland T.A., Keseler I.M., Kothari A., Kubo A. (2014). The MetaCyc database of metabolic pathways and enzymes and the BioCyc collection of Pathway/Genome Databases. Nucleic Acids Res..

[21] Parks D.H., Tyson G.W., Hugenholtz P., Beiko R.G. (2014). STAMP: Statistical analysis of taxonomic and functional profiles. Bioinformatics.

[22] Singh R.P., Halaka D.A., Hayouka Z., Tirosh O. (2020). High-Fat Diet Induced Alteration of Mice Microbiota and the Functional Ability to Utilize Fructooligosaccharide for Ethanol Production. Front. Cell. Infect. Microbiol..

[23] Amabebe E., Robert F.O., Agbalalah T., Orubu E.S.F. (2020). Microbial dysbiosis-induced obesity: Role of gut microbiota in homoeostasis of energy metabolism. Br. J. Nutr..

[24] Yoshida N., Yamashita T., Osone T., Hosooka T., Shinohara M., Kitahama S., Sasaki K., Sasaki D., Yoneshiro T., Suzuki T. (2021). Bacteroides spp. promotes branched-chain amino acid catabolism in brown fat and inhibits obesity. iScience.

[25] Jones R.B., Alderete T.L., Kim J.S., Millstein J., Gilliland F.D., Goran M.I. (2019). High intake of dietary fructose in overweight/obese teenagers associated with depletion of Eubacterium and Streptococcus in gut microbiome. Gut Microbes.

[26] Kong C., Gao R., Yan X., Huang L., Qin H. (2019). Probiotics improve gut microbiota dysbiosis in obese mice fed a high-fat or high-sucrose diet. Nutrition.

[27] Wang S., Huang M., You X., Zhao J., Chen L., Wang L., Luo Y., Chen Y. (2018). Gut microbiota mediates the anti-obesity effect of calorie restriction in mice. Sci. Rep..

[28] Shi Z., Fang Z.Y., Gao X.X., Yu H., Zhu Y.W., Ouyang H.L., Song Y.X., Du X.L., Wang Z., Li X.W. (2021). Nuciferine improves high-fat diet-induced obesity via reducing intestinal permeability by increasing autophagy and remodeling the gut microbiota. Food Funct..

[29] Sun X., Zhao H., Liu Z., Sun X., Zhang D., Wang S., Xu Y., Zhang G., Wang D. (2020). Modulation of Gut Microbiota by Fucoxanthin During Alleviation of Obesity in High-Fat Diet-Fed Mice. J. Agric. Food Chem..

[30] Kameyama K., Itoh K. (2014). Intestinal colonization by a Lachnospiraceae bacterium contributes to the development of diabetes in obese mice. Microbes Environ..

[31] Palmas V., Pisanu S., Madau V., Casula E., Deledda A., Cusano R., Uva P., Vascellari S., Loviselli A., Manzin A. (2021). Gut microbiota markers associated with obesity and overweight in Italian adults. Sci. Rep..

[32] Zhou Q., Zhang Y., Wang X., Yang R., Zhu X., Zhang Y., Chen C., Yuan H., Yang Z., Sun L. (2020). Gut bacteria Akkermansia is associated with reduced risk of obesity: Evidence from the American Gut Project. Nutr. Metab..

[33] Denou E., Marcinko K., Surette M.G., Steinberg G.R., Schertzer J.D. (2016). High-intensity exercise training increases the diversity and metabolic capacity of the mouse distal gut microbiota during diet-induced obesity. Am. J. Physiol. Endocrinol. Metab..

[34] Mills E.L., Pierce K.A., Jedrychowski M.P., Garrity R., Winther S., Vidoni S., Yoneshiro T., Spinelli J.B., Lu G.Z., Kazak L. (2018). Accumulation of succinate controls activation of adipose tissue thermogenesis. Nature.

[35] Ma Q., Zhou X., Sun Y., Hu L., Zhu J., Shao C., Meng Q., Shan A. (2020). Threonine, but Not Lysine and Methionine, Reduces Fat Accumulation by Regulating Lipid Metabolism in Obese Mice. J. Agric. Food Chem..

[36] Hernandez-Castillo C., Shuck S.C. (2021). Diet and Obesity-Induced Methylglyoxal Production and Links to Metabolic Disease. Chem. Res. Toxicol..

[37] Matafome P., Sena C., Seica R. (2013). Methylglyoxal, obesity, and diabetes. Endocrine.

[38] Knapen M.H.J., Jardon K.M., Vermeer C. (2018). Vitamin K-induced effects on body fat and weight: Results from a 3-year vitamin K2 intervention study. Eur. J. Clin. Nutr..

[39] Hersoug L.G., Moller P., Loft S. (2018). Role of microbiota-derived lipopolysaccharide in adipose tissue inflammation, adipocyte size and pyroptosis during obesity. Nutr. Res. Rev..

[40] Zhao M., Chen X. (2015). Effect of lipopolysaccharides on adipogenic potential and premature senescence of adipocyte progenitors. Am. J. Physiol. Endocrinol. Metab..

