# Supplementary material for: Collagen Peptide Exerts an Anti-Obesity Effect by Influencing the Firmicutes/Bacteroidetes Ratio in the Gut
Source: Nutrients. 2023 Jun 2;15(11):2610. doi: 10.3390/nu15112610 (PMC10255498; doi:10.3390/nu15112610)
Supplement: Supplementary file 1 [file nutrients-15-02610-s001.zip › nutrients-2431110-supplementary.pdf]

Table S1. Distribution of molecular weight for collagen peptide.

**M.W. (Dalton)**	**M.W. Distribution (%)**
10,000 over	0.0
5,000 ~ 10,000	0.2
1,000 ~ 5,000	19.8
500 ~ 1,000	50.8
500 below	29.2
Total	100%

Table S2. Distribution of molecular weight for collagen peptide.

Amino acids	Contents (%)
**Aspartic acid**	4.68
**Threonine**	2.76
**Serine**	3.12
**Glutamic acid**	9.11
**Proline**	12.59
**Glycine**	21.71
**Alanine**	9.56
**Valine**	2.02
**Isoleucine**	0.96
**Leucine**	2.11
**Tyrisine**	0.67
**Phenylalanine**	1.63
**Histidine**	1.65
**Lysine**	3.08
**Arginine**	6.86
**Cystine**	0.13
**Methionine**	1.67
**Hydroxy-Proline**	11.47
**Hydroxy-Lysine**	0.74

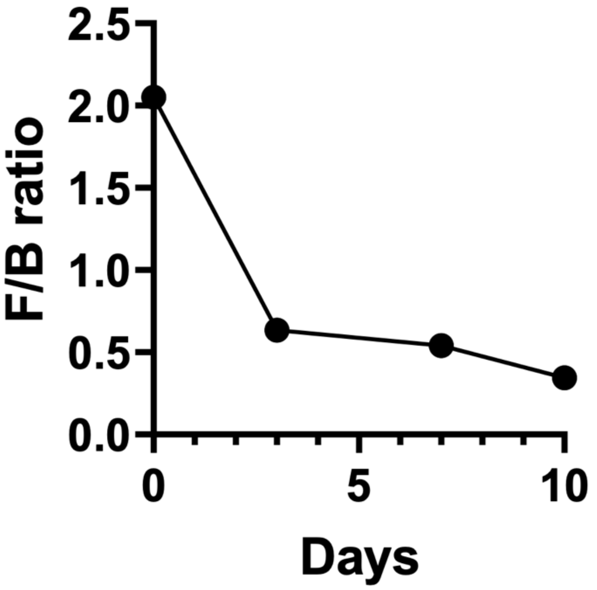

Figure S1. Changes in F/B ratio in the gut microbiome over time. The change in the F/B ratio in the gut microbiome was confirmed from the 3rd day of collagen peptide administration.

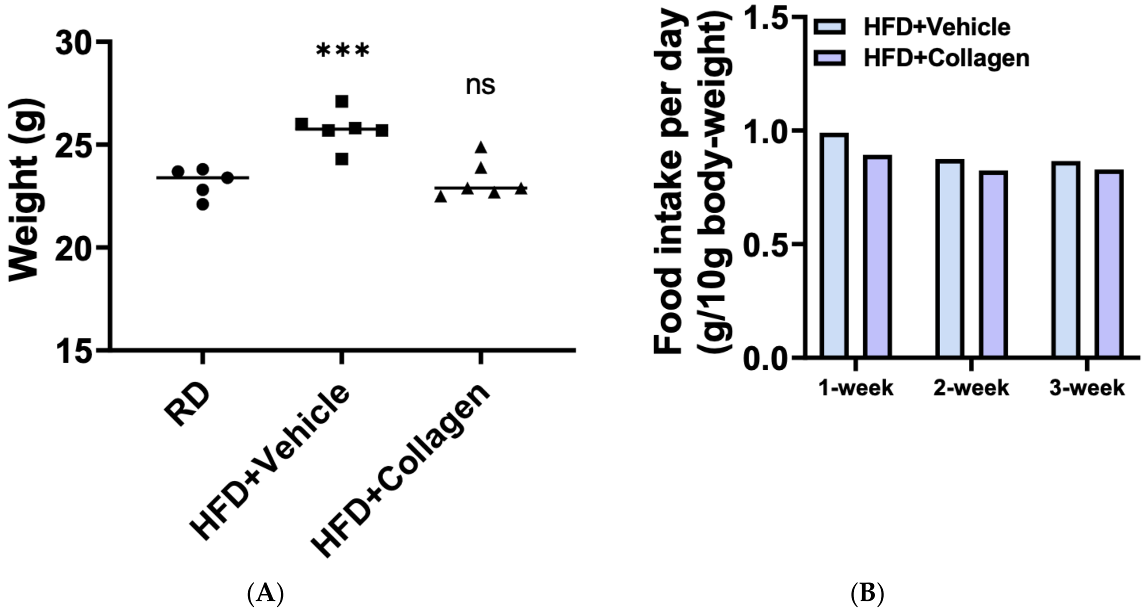

Figure S2. Comparison of body weights for 3 weeks in the three groups (A). Comparison of changes in daily food intake between the two groups for 3 weeks (B). Unpaired t-tests (two-tailed) were used to analyze variations between the two groups. ***p <0.001.

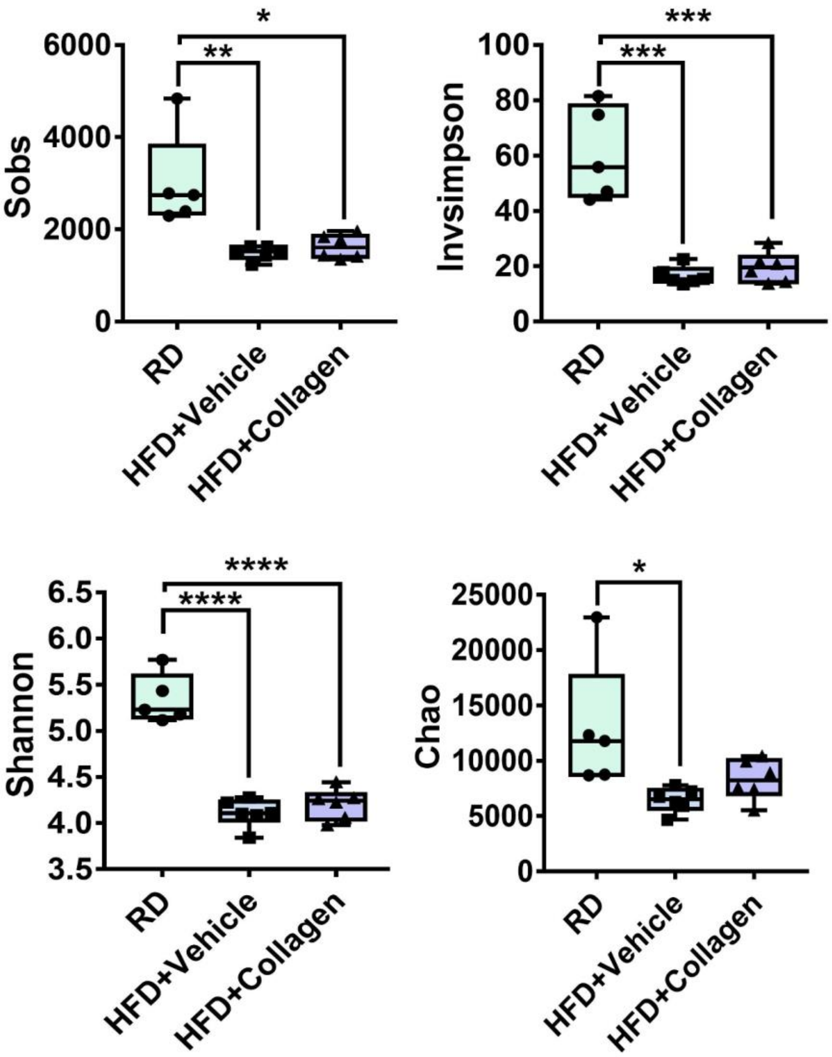

Figure S3. Alpha diversity. Sobs, Shannon, Invsimpson, and Chao indices reflect the differences in microbiota richness and evenness among samples. Unpaired t-tests (two-tailed) were used to analyze variation between the two groups. *p <0.05, **p <0.01, ***p <0.001, ****p<0.0001.

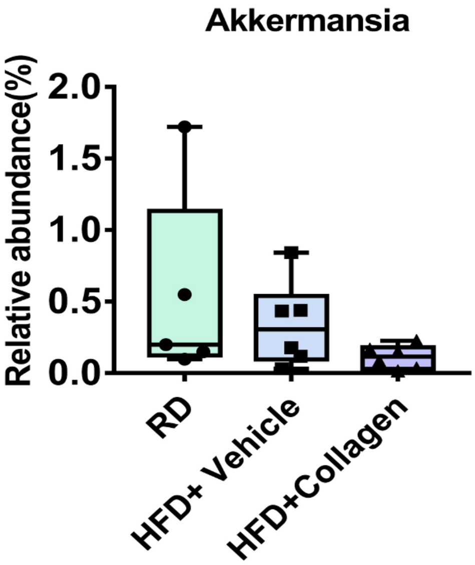

Figure S4. Comparison of relative abundance in the genus *Akkermansia* known to have anti-obesity effects. An unpaired t-test (two-tailed) was used to analyze group variations. There was no significant difference.

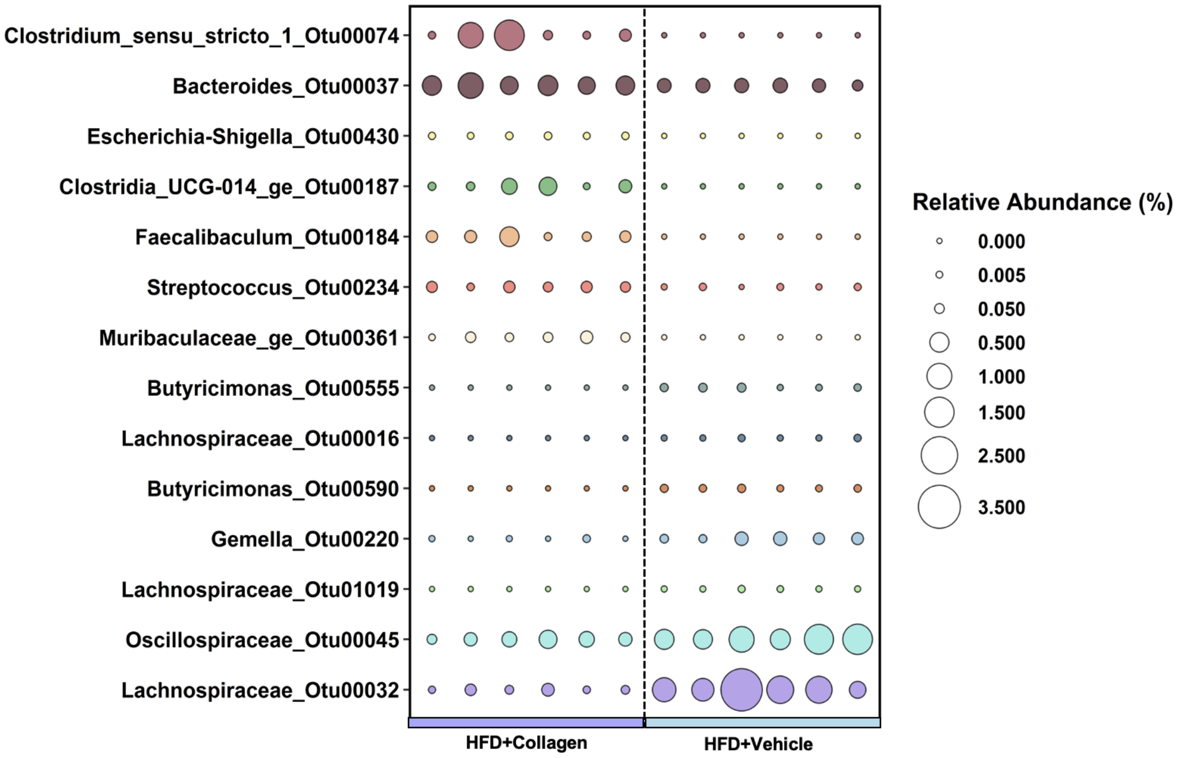

Figure S5. Bubble plot of the average relative abundance of OTUs significantly differed between the two groups. Circle size indicated the relative abundance from 0 to 3.5%.

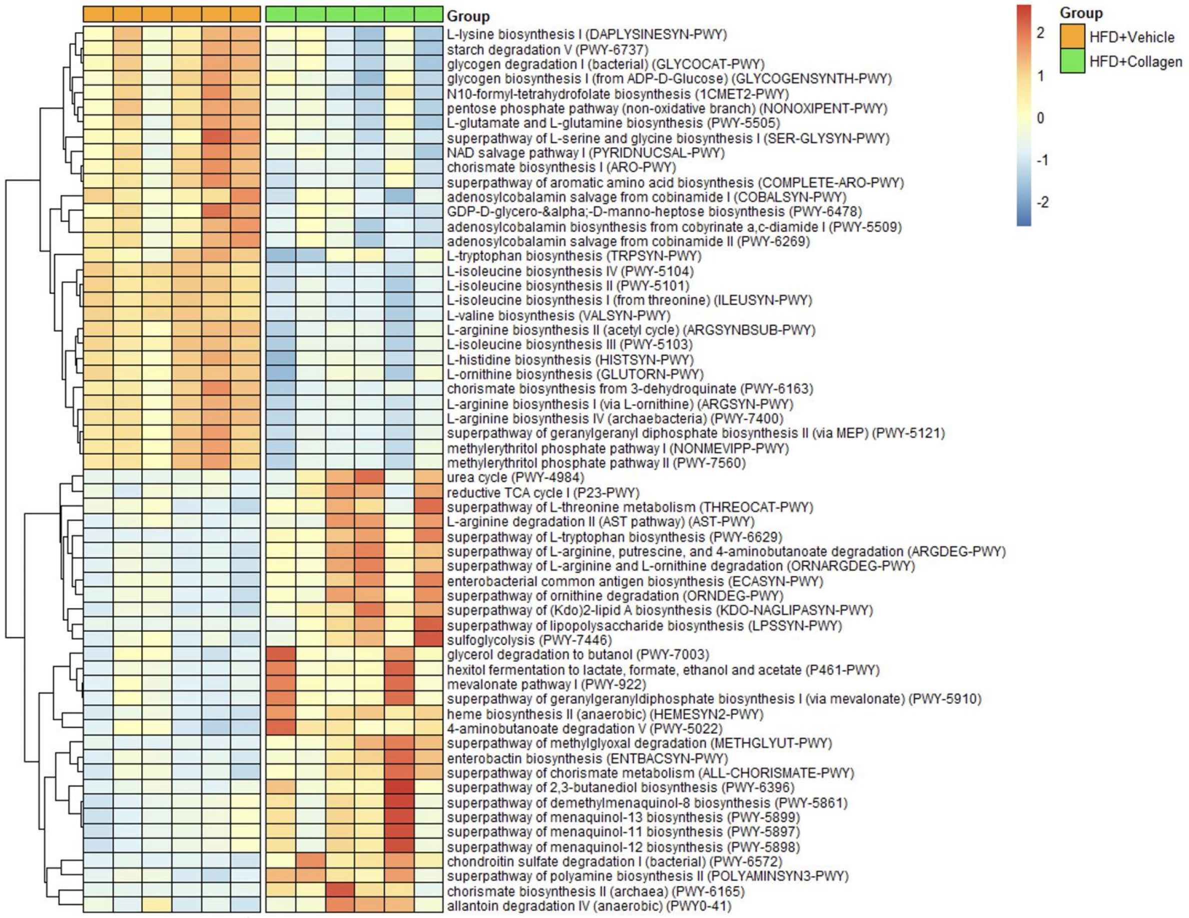

Figure S6. Heatmap visualization of significantly different functional profiles inferred by PICRUST2 according to the presence or absence of collagen peptide. Heatmaps were drawn with normalized relative abundances in all the pathways in the two groups. Red colors represent higher abundance, and blue colors lower abundance.
